# Crystal Structure of L-Histidinium 2-Nitrobenzoate

**DOI:** 10.1155/2012/463183

**Published:** 2012-03-25

**Authors:** Subramanian Natarajan, Kalimuthu Moovendaran, Jeyaperumal Kalyana Sundar, Krishnan Ravikumar

**Affiliations:** ^1^Department of Physics, Madurai Kamaraj University, Madurai 625 021, India; ^2^Department of Physics, Sethu Institute of Technology, Pulloor, Kariapatti 626115, India; ^3^Indian Institute of Chemical Technology, Hyderabad 500 007, India

## Abstract

A new nonlinear optical organic compound, namely, L-histidinium 2-nitrobenzoate (abbreviated as LH2NB (I); ([C_6_H_10_N_3_O_2_]^+^ [C_7_H_4_NO_4_]^−^)), was synthesized. The molecular structure of LH2NB (I) was elucidated using single crystal X-ray diffraction technique. The second harmonic generation (SHG) efficiency of this compound is about two times that of the standard potassium dihydrogen phosphate crystals.

## 1. Introduction

Recently, considerable efforts are being made to design new noncentrosymmetric crystal structures by combining amino acids with various interesting organic and inorganic matrices to produce compounds for nonlinear optical (NLO) applications. A number of L-histidine compounds exhibiting the NLO behaviour, namely, L-histidine acetate [1], L-histidine chloride monohydrate [2], L-histidine tetrafluoroborate [3], L-histidine hydrochloride monohydrate [4], L-histidine hydrofluoride dihydrate [5], L-histidine bromide [6], and L-histidinium trichloroacetate [7], were reported earlier. The crystal growth and characterization of L-histidinium trifluoroacetate and L-histidine nitrate were reported from this laboratory [8, 9], recently. In this paper, another new compound possessing the NLO property, namely, L-histidinium 2-nitrobenzoate [LH2NB, (I)] is reported. To our knowledge, (I) is the first reported compound of an amino acid with 2-nitrobenzoic acid. The details regarding the preparation, crystal structure, hydrogen bonding, and SHG efficiency of the title compound are discussed.

## 2. Experimental Procedures

### 2.1. Synthesis and Crystallization of LH2NB

**Figure 1:**
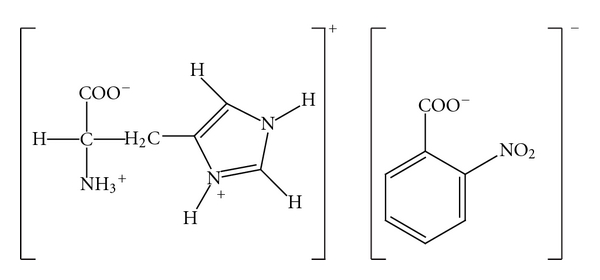
The chemical structure of (I).

The starting compounds, namely, L-histidine (Loba Chemie, 99%) and 2-nitrobenzoic acid (Alfa Aesar, 95%) were used without further purification. L-histidine and 2-nitrobenzoic acid were mixed in the stoichiometric ratio, in 1 : 1 proportions and dissolved in distilled water. The resultant mixture was stirred continuously to obtain a homogeneous solution, filtered and kept undisturbed for crystallization to take place. Good quality single crystals of the title compound were obtained after about a week's time. The chemical structure of the compound is shown in Figure 1.

### 2.2. Crystal Structure Determination

Three-dimensional intensity data for a crystal of (I) were collected on a Bruker SMART APEX CCD area-detector diffractometer using graphite-monochromated MoK_*α*_ radiation (0.71073 Å). The crystal structure was solved by direct methods using SHELXS-97 [10]. Full-matrix least-squares refinement and subsequent Fourier synthesis procedures were performed by using SHELXL-97 [10]. The hydrogen atoms attached to the C atoms were positioned with idealized geometry and refined using a riding model [C–H = 0.93–0.97 Å, U_iso_ = 1.2 U_eq_ (parent C atom)]. All the other hydrogen atoms participating in N–H bonding were located from difference Fourier map and restrained to a distance of 0.86 Å. In the absence of significant anomalous scattering effects, Friedel pairs (1130) were merged. The absolute configuration of L-histidinium 2-nitrobenzoate was known in advance. Successive refinements based on *F* ^2^ led to an *R* value of 0.0254. The crystal data, structure solution, and refinement parameters are listed in Table 1.

Crystallographic data (excluding structure factors) for the structure of compound (I) reported in this paper have been deposited with the Cambridge Crystallographic Data Centre as supplementary publication no. CCDC 857702. Copies of the data can be obtained, free of charge, on application to CCDC, 12 Union Road, Cambridge CB2 1 EZ, UK (fax: +44-(0)1223-336033 or email: deposit@ccdc.cam.ac.uk).

### 2.3. Second Harmonic Generation (SHG) Efficiency

A preliminary study of the powder SHG was made with a laser beam of wavelength 1064 nm, using Kurtz and Perry technique [11]. The beam from a Q switched Nd: YAG laser had an energy of 3.9 mJ/pulse, pulse width of 8 ns, and the repetition rate being 10 Hz. The crystals were ground to a uniform particle size of about 125–150 *μ*m and then packed in capillaries of uniform bore and exposed to the laser radiation. A powder of potassium dihydrogen phosphate (KDP), with the same particle size, was used as a reference. The output from the sample was monochromated to collect only the second harmonic (*λ* = 532 nm), eliminating the fundamental, and the intensity was measured using a photomultiplier tube. It was found that the SHG conversion efficiency for the compound (I) is about two times that of the standard KDP crystals.

## 3. Results and Discussions

### 3.1. Structure Description

**Figure 2:**
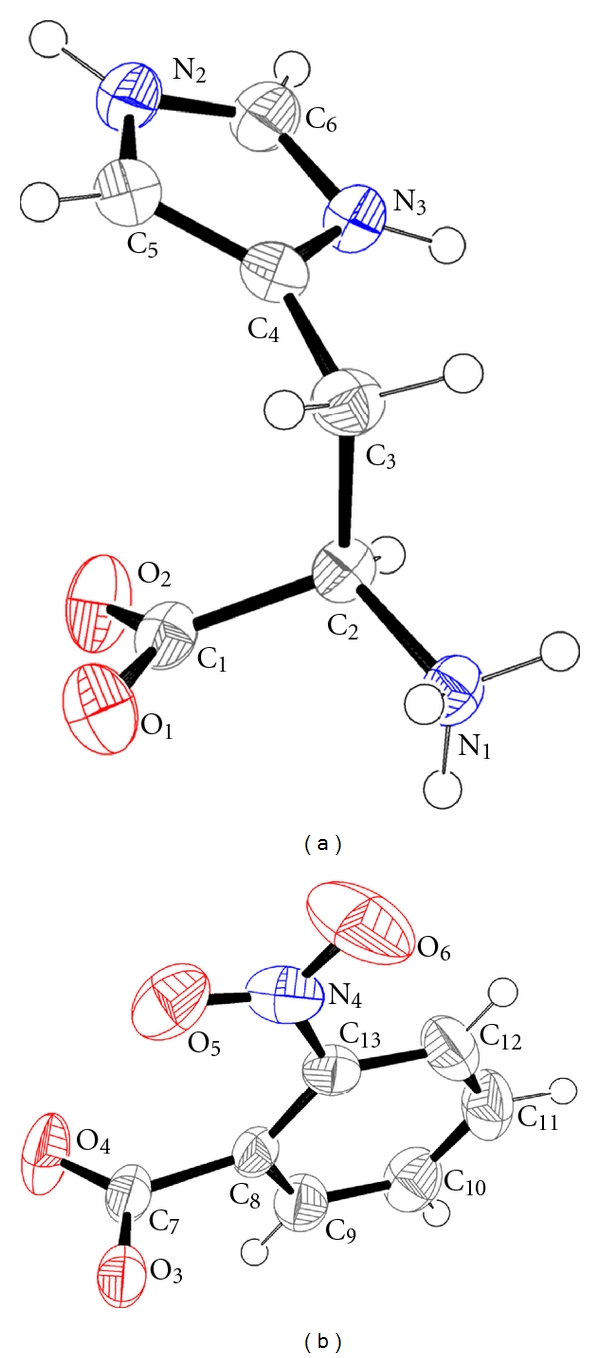
The molecular structure of (I) showing the atom numbering scheme. Displacement ellipsoids are drawn at 50% probability level, using ORTEP-3. Hydrogen atoms are drawn as spheres of arbitrary size.

The molecular structure of the compound (I) with atom numbering scheme is shown in Figure 2. The asymmetric part of the unit cell contains an L-histidinium cation and a nitrobenzoate anion. The histidine molecule exists as histidinium ion due to the protonation at the N atom of the imidazole ring. The 2-nitrobenzoic acid exists as nitrobenzoate since the proton gets transferred to the amino acid. Selected bond lengths, bond and torsion angles of (I) are listed in Table 2.

**Figure 3:**
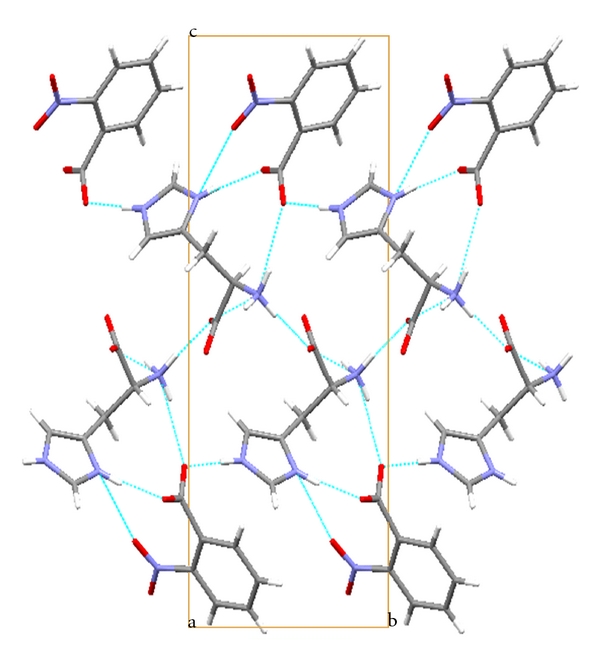
The packing diagram of LH2NB viewed down the *a*-axis. Hydrogen bonds are shown as dashed lines.

The amino and carboxylic groups of histidine are twisted by an angle (C1–C2–C3–C4) of −71.12 (2)° (Table 2), while this angle is −179.13(2)° in the structure of L-histidine [12]. This type of rotation is a common feature in histidine-carboxylic acid structures [1, 8, 13–15]. The bond angles in the amino and carboxyl groups are normal as observed in the structure of L-histidine [12]. The plane of the carboxylate group of histidinium is twisted from its normal position [12], which may be due to the presence of the intermolecular N–H*⋯*O bonds (Figure 3) from the nearby histidinium ions.

The benzene ring of nitrobenzoate is nearly planar with the r.m.s. deviation being only 0.010 (2) Å. The nitro and carboxylate groups of the nitrobenzoate are twisted with respect to the planar benzene ring, as evident from the values of the torsion angles [16]. The planar six membered and five membered rings in nitrobenzoate and histidinium, respectively, are nearly perpendicular [82.99°] to each other. Excepting O6, all the other oxygen atoms act as acceptors (Figure 3) in the formation of N–H*⋯*O and C–H*⋯*O hydrogen bonds (Table 3).

### 3.2. Structural Features Responsible for the Origin of SHG in the Compound (I)

**Figure 4:**
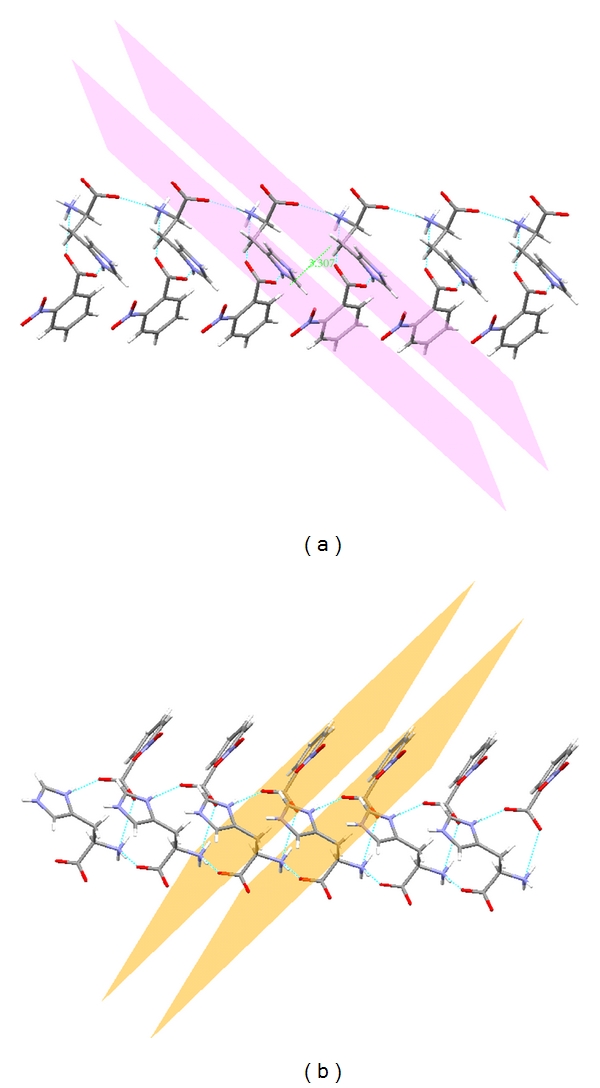
(a) The histidinium is stacked as arrays along the *a*-axis. (b) The 2-nitrobenzoate is stacked as arrays along the *a*-axis.

The level of SHG response of a material is inherently dependent upon its structural attributes. On a molecular scale, the extent of charge transfer (CT) across the NLO chromophore determines the level of SHG output; the greater the CT, the larger the SHG output. The presence of intermolecular interactions, such as hydrogen bonds, can extend this level of CT into the supramolecular realm, owing to their electrostatic and directed nature, thereby enhancing the SHG response [17, 18]. In LH2NB, the network of N–H*⋯*O hydrogen bonds running along the *a*-axis links the cationic histidinium ions (Figure 4(a)). Further, the benzoates are interconnected by N–H*⋯*O hydrogen bond to the histidinium molecule. The interconnected molecules of LH2NB are stacked as arrays along the *a*-axis (Figures 4(a) and 4(b)) and further stabilized by other N–H*⋯*O bonds and weak interactions. 

The interplanar distances between the two six membered rings (in 2-nitrobenzoates) and the two five membered rings (in the amino acids) have the values of 3.480 Å and 3.307 Å, respectively, which fall in the category of *π*-*π* stacking. Such parallel and close stacking is very favorable for the promotion of CT through the lattice. The large SHG efficiency (about two times that of the standard KDP) of the compound (I) possibly arises due to (i) the large number of hydrogen bonds and (ii) the close stacking of histidinium cations and 2-nitrobenzozte anions, in the structure [19]. 

## 4. Conclusions

The crystals of a new NLO material from the amino acid family, namely, L-histidinium 2-nitrobenzoate (LH2NB), were grown using slow evaporation technique. The crystal structure of LH2NB was elucidated using single crystal X-ray diffraction methods. The proton from 2-nitrobenzoic acid is transferred to the L-histidine, forming L-histidinium 2-nitrobenzoate. The SHG efficiency of this material was measured using Kurtz and Perry method and found to be about two times that of the standard KDP crystals. The large number of hydrogen bonds, the *π*-*π* stacking of L-histidinium anions and 2-nitrobenzoate cations and the presence of strong electron acceptors and electron donors are, in part, the reasons for the large SHG efficiency possessed by this material.

## Figures and Tables

**Table 1:** The crystal data, structure solution, and refinement parameters for (I).

Empirical formula	C_13_H_14_N_4_O_6_
Formula weight	322.28
Temperature	294 (2) K
Crystal system, space group	Monoclinic, P2_1_
Unit cell dimensions	*a* = 5.147 (3) Å; *b* = 7.228 (5) Å *c* = 18.887 (1) Å; *β* = 92.72 (1)°
Volume	701.8 (8) Å^3^
*Z*, calculated density	2, 1.525 kg/m^3^
Absorption coefficient	0.123 mm^−1^
F(000)	336
Crystal size	0.24 × 0.25 × 0.26 mm^3^
Theta range	2.16° to 24.99°
Limiting indices	−6 ≤ *h* ≤ 6, − 8 ≤ *k* ≤ 8, − 22 ≤ *l* ≤ 22
Reflections collected/unique	6680/1347 [*R*(int) = 0.0176]
Completeness to *θ* = 25°	99.9%
Data/restraints/parameters	1347/6/228
Goodness-of-fit on *F * ^2^	1.068
Final *R* indices [*I* > 2*σ* (I)]	*R*_1_ = 0.0254, *wR*_2_ = 0.0703
*R* indices (all data)	*R*_1_ = 0.0255, *wR*_2_ = 0.0705
Largest diff. peak and hole	0.166 and −0.141 e·Å^−3^

**Table 2:** Selected bond lengths (Å), bond and torsion angles (°) of (I).

C1–O1	1.224(2)	C6–N2	1.316 (3)
C1–O2	1.266 (2)	C6–N3	1.321 (3)
C1–C2	1.530 (2)	C7–O4	1.234 (2)
C2–N1	1.483 (2)	C7–O3	1.253 (2)
C2–C3	1.533 (2)	C7–C8	1.523 (3)
C3–C4	1.485 (3)	C13–N4	1.459 (3)
C4–C5	1.354 (3)	N4–O6	1.221 (3)
C4–N3	1.371 (3)	N4–O5	1.218 (3)
C5–N2	1.370 (3)		
O1–C1–O2	126.5 (2)	C9–C8–C13	116.3 (2)
O1–C1–C2	118.0 (2)	C9–C8–C7	116.6 (2)
O2–C1–C2	115.5 (2)	C13–C8–C7	127.0 (2)
N1–C2–C1	110.7 (1)	C8–C9–C10	122.2 (2)
N1–C2–C3	107.8 (1)	C11–C10–C9	119.7 (2)
C1–C2–C3	110.2 (2)	C12–C11–C10	119.7 (2)
C4–C3–C2	112.2 (2)	C11–C12–C13	119.8 (2)
C5–C4–N3	106.3 (2)	C12–C13–C8	122.2 (2)
C5–C4–C3	132.3 (2)	C12–C13–N4	117.7 (2)
N3–C4–C3	121.4 (2)	C8–C13–N4	120.1 (2)
C4–C5–N2	107.3 (2)	C6–N2–C5	108.7 (2)
N2–C6–N3	108.7 (2)	C6–N3–C4	109.1 (2)
O4–C7–O3	126.9 (2)	O5–N4–O6	123.5 (3)
O4–C7–C8	116.9 (2)	O5–N4–C13	17.9 (2)
O3–C7–C8	115.9 (2)	O6–N4–C13	118.6 (2)
O1–C1–C2–N1	39.6 (2)	O4–C7–C8–C9	−75.8 (3)
C1–C2–C3–C4	−71.1 (2)	O3–C7–C8–C9	98.7 (2)
O3–C7–C8–C13	−78.5 (3)	O4–C7–C8–C13	107.0 (2)
C7–C8–C13–N4	−9.6 (3)	C12–C13–N4–O5	170.0 (2)
O1–C1–C2–C3	−79.6 (2)	C8–C13–N4–O5	−6.8 (3)
O2–C1–C2–C3	97.2 (2)	C12–C13–N4–O6	−8.8 (3)
C2–C3–C4–C5	104.4 (2)	C8–C13–N4–O6	174.5 (2)

**Table 3:** Hydrogen-bond geometry of (I) (Å, °).

D–H*···*A	D–H	H*⋯*A	D*⋯*A	<(D–H*⋯*A)
N1–H1N*⋯*O2^i^	0.89	1.97	2.847 (2)	166 (2)
N1–H2N*⋯*O2^ii^	0.90	1.84	2.738 (2)	174 (2)
N1–H3N*⋯*O3^iii^	0.91	2.18	3.020 (2)	154 (2)
N2–H4N*⋯*O3^iv^	0.87	1.81	2.685 (3)	174 (3)
N3–H5N*⋯*O4^iii^	0.85	1.88	2.712 (3)	164 (3)
N3–H5N*⋯*O5^v^	0.85	2.59	3.019 (3)	112 (2)
C5–H5 *⋯*O1^vi^	0.93	2.41	3.052 (2)	126 (2)
C6–H6*⋯*O5^v^	0.93	2.36	2.931 (3)	119 (2)

^i^−1 + *x*, *y*, *z*; ^ii^2 − *x*, 1/2 + *y*, 1 − *z*; ^iii^ *x*, 1 + *y*, *z*; ^iv^1 + *x*, *y*, *z*; ^v^1 + *x*, 1 + *y*, *z*; ^vi^2 − *x*, −1/2 + *y*, 1 − *z*.
